# Ten-Year Experience with Endomyocardial Biopsy after Orthotopic Heart Transplantation: Comparison between Trans-Jugular and Trans-Femoral Approach

**DOI:** 10.3390/jcdd11040115

**Published:** 2024-04-03

**Authors:** Antonella Galeone, Annalisa Bernabei, Gabriele Pesarini, Marcello Raimondi Lucchetti, Francesco Onorati, Giovanni Battista Luciani

**Affiliations:** 1 Department of Surgery, Dentistry, Pediatrics and Gynecology, Division of Cardiac Surgery, University of Verona, 37126 Verona, Italy; 2 Department of Thoracic and Cardiovascular Surgery, Heart Vascular and Thoracic Institute, Cleveland Clinic, Cleveland, OH 44195, USA; 3 Division of Cardiology, Azienda Ospedaliera Universitaria Integrata, 37126 Verona, Italy

**Keywords:** endomyocardial biopsy, heart transplantation, jugular vein

## Abstract

Background: Endomyocardial biopsy (EMB) is considered the gold standard for monitoring allograft rejection after heart transplantation. EMB is an invasive procedure that may be performed via a trans-jugular or a trans-femoral approach with a complication rate reported as less than 6%. The aim of this study was to evaluate the complication rate after EMBs in heart recipients and to compare the results of EMBs performed via a trans-jugular or a trans-femoral approach. Methods: Medical records of heart recipients undergoing EMBs between January 2012 and December 2022 were retrospectively reviewed. EMB-related complications were classified as major (death, pericardial effusion, hemopericardium, cardiac tamponade requiring a pericardiocentesis or an urgent cardiac surgery, ventricular arrythmias, permanent atrio-ventricular block requiring permanent pacing, hemothorax, pneumothorax and retroperitoneal bleeding) and minor (de novo tricuspid regurgitation, arrhythmias, coronary artery fistula, vascular access site complications). Results: A total of 1698 EMBs were performed during the study period at our institution in 212 heart recipients. There were 927 (55%) EMBs performed through a trans-jugular approach (TJ group) and 771 (45%) EMBs performed through a trans-femoral approach (TF group). A total of 60 (3.5%) complications were recorded, including nine (0.5%) major complications (six cardiac tamponades, two pneumothorax and one retroperitoneal bleeding) and 51 (3%) minor complications (seven coronary fistulae, five de novo tricuspid regurgitation, four supraventricular arrythmias and thirty-five vascular access site complications). No difference was found in total (38 [4%] vs. 22 [3%]; *p* = 0.16) and major (6 [1%} vs. 3 [0.4%]; *p* = 0.65) complications (32 [3%] vs. 19 [2%]; *p* = 0.23) between the TJ group and the TF group. No difference was found in male sex, age at time of EMB and time from HT between complicated and not complicated EMBs. Conclusions: EMBs represent a safe procedure with a low risk of complications. In our experience, EMBs performed via a trans-jugular approach are as safe as the trans-femoral approach.

## 1. Introduction

Heart transplantation (HT) represents the treatment of choice for patients with end-stage heart failure, providing improved survival and quality of life in these patients [1]. Despite significant advances in immunosuppressive therapies and continuous surveillance, allograft rejection and graft dysfunction caused by rejection remain a leading cause of morbidity and mortality and represent important limitations for long-term survival after HT. Acute rejection episodes account for 8% of deaths within the first three years after transplantation [2]. Later, the incidence and impact on death decreases markedly; however, acute rejection triggers progression of cardiac allograft vasculopathy which significantly affects graft performance and survival [3]. Endomyocardial biopsy (EMB) is considered the gold standard for monitoring acute rejection and for the diagnosis of both acute cellular rejection (ACR) and antibody-mediated rejection (AMR). The first cardiac biopsy was performed through a transthoracic needle approach by Sutton in 1956 [4]. Konno and Sakakibara first reported a percutaneous EMB procedure in 1962 using a flexible bioptome with sharpened cusps that allowed an EMB via pinching [5]. Caves modified the Konno–Sakakibara bioptome in 1973 in order to perform the procedure through the internal jugular vein [6], and the Stanford–Caves bioptome is still used for EMB. In 1984, the femoral vein approach was also proposed by Anderson et al. [7].

The frequency of surveillance EMBs is typically greatest during the first 3 to 6 months after transplantation, the time at which ACR is most common. Although there are internationally accepted grading systems for ACR and AMR [8], EMBs may have a low sensitivity and are associated with significant interobserver variability in histopathologic interpretation [9]. EMBs may cause patient discomfort, thus limiting the temporal frequency of assessments, and its routine use can also lead to the occlusion of access points and damage to the allograft. EMBs are an invasive procedure that may be performed through a trans-jugular (TJ) or a trans-femoral (TF) approach with a complication rate reported as less than 6% [10]. To date, no study has compared the incidence of EMB-related complications between the TJ and the TF approach. Thus, the aim of this study was to evaluate the incidence of procedure-related complications in heart recipients and to compare the results of EMBs performed either via a TJ or a TF approach.

## 2. Materials and Methods

This study was conducted in accordance with the Declaration of Helsinki and ethical approval was waived by the local Ethics Committee due to the observational and retrospective nature of this study.

The institutional protocol for allograft rejection surveillance requires EMBs to be performed weekly during the first month after transplantation, biweekly during the second and third months, at the fourth, fifth, sixth, ninth and twelfth month after transplantation, and annually following the first year. The protocol for allograft rejection surveillance did not change during the study period.

Additional EMBs were performed in case of clinical suspicion of acute allograft rejection or after an episode of acute allograft rejection to monitor the efficacy of the immunosuppressive therapy. Biopsies were evaluated for rejection using the revised International Society of Heart and Lung Transplantation criteria [8].

High-dose corticosteroids were the first-line therapy for acute cellular allograft rejection with a grade greater than 1R. All consecutive patients who received at least one EMB for allograft rejection surveillance after HT at our institution between January 2012 and December 2022 were included in the study. Medical records of all patients were retrospectively reviewed, and patients were further stratified by the access site of EMB. EMBs were performed either through the right jugular vein or the femoral vein. We did not routinely use ultrasound guidance for venous puncture. Transjugular EMBs were performed by experienced cardiac surgeons, while TF EMBs were performed by experienced cardiologists. A single-use 50-cm Novatome bioptome (Sholten Surgical Instruments, Inc., Lodi, CA, USA) requiring a 9-F sheath was used for trans-jugular vein EMBs, while a 104-cm Bipal 7 bioptome (Cordis Corp, Miami Lakes, FL, USA) requiring a 7-F sheath was used for trans-femoral vein EMBs. Fluoroscopy-guided EMBs were procured in the septal apical region of the right ventricle according to the guidelines for allograft rejection surveillance [11]. All EMBs were performed under local anesthesia and at least three samples were obtained in all patients for histopathological analysis. Non-invasive parameters were continuously monitored during the procedure. Following their EMBs, all patients underwent 12-lead electrocardiography, chest radiograph and transthoracic echocardiography to assess the appearance of procedure-related complications. EMB-related complications were classified as major (death, advanced cardiac life support, pericardial effusion/hemopericardium/cardiac tamponade requiring a pericardiocentesis or an urgent cardiac surgery, ventricular arrythmias, permanent atrio-ventricular block requiring permanent pacing, hemothorax, pneumothorax and vascular access site complication requiring surgery and/or transfusions as retroperitoneal bleeding) and minor (de novo moderate to severe tricuspid regurgitation, coronary artery fistula, supraventricular arrhythmias, vascular access site complications as hematoma, pseudoaneurysm, arteriovenous fistula and accidental arterial puncture). Categorical variables were expressed as number and percentages and compared with χ^2^ test. Continuous variables with a skewed distribution are presented as median and interquartile range and compared with the Mann–Whitney U test. A two-tailed *p* value < 0.05 was taken to indicate statistical significance. Statistical analysis was performed using Sigmaplot version 15.0 (Systat Software Inc., San Jose, CA, USA).

## 3. Results

**Figure 1:**
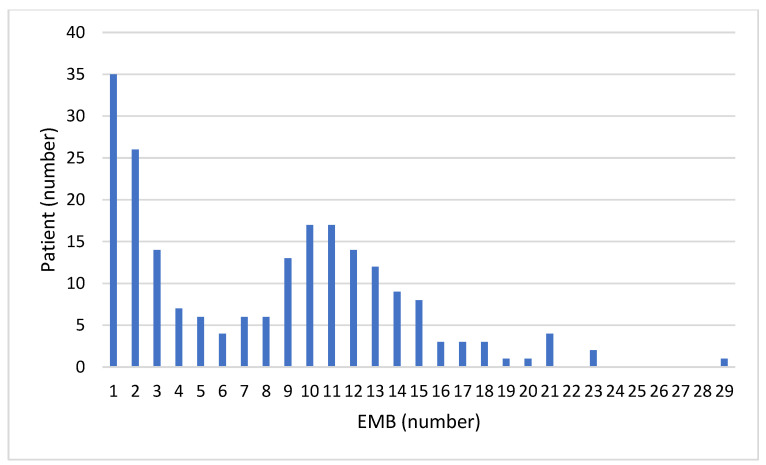
Distribution graph indicating the actual number of endomyocardial biopsies (EMBs) per patient.

Two-hundred-twelve heart recipients received at least one EMB for allograft rejection surveillance during the study period at our institution and were included in the study. The clinical characteristics of heart recipients included in the study are illustrated in Table 1. The cohort was composed of 54 (25%) females and 158 (75%) males; median age at time of HT was 57 years and the main indications for HT were dilated (n = 86, 41%), ischemic (n = 85, 40%) and valvular (n = 11, 5%) cardiomyopathy. During the study period, the median number of EMBs was nine (2–12)for each patient (Figure 1).

**Figure 2:**
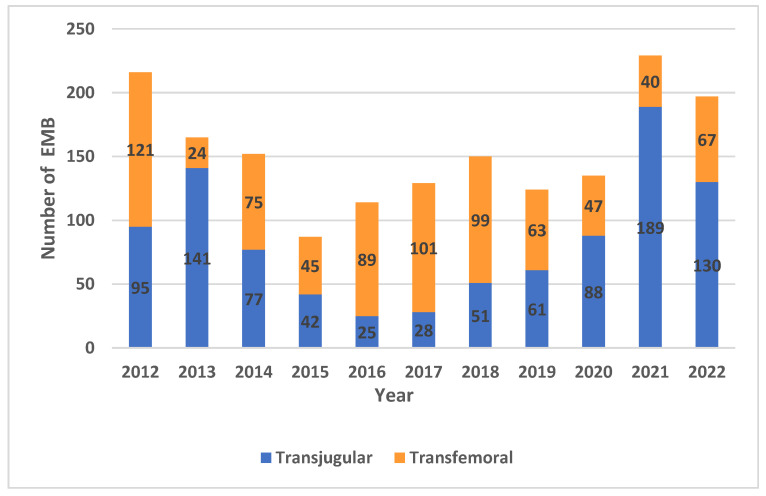
Number of endomyocardial biopsies (EMBs) performed each year at our institution from 2012 to 2022.

**Figure 3:**
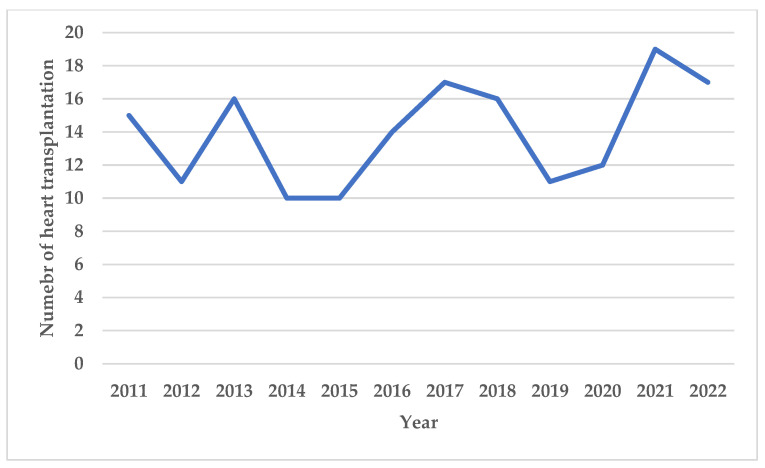
Number of heart transplantations performed each year at our institution from 2011 to 2022.

A total of 1698 EMBs were performed during the study period through either a TJ (n = 927, 55%) or a TF (n = 771, 45%) approach. The number of EMBs performed each year from 2012 to 2022 according to the access site is illustrated in Figure 2. The number of HTs performed each year is illustrated in Figure 3.

**Figure 4:**
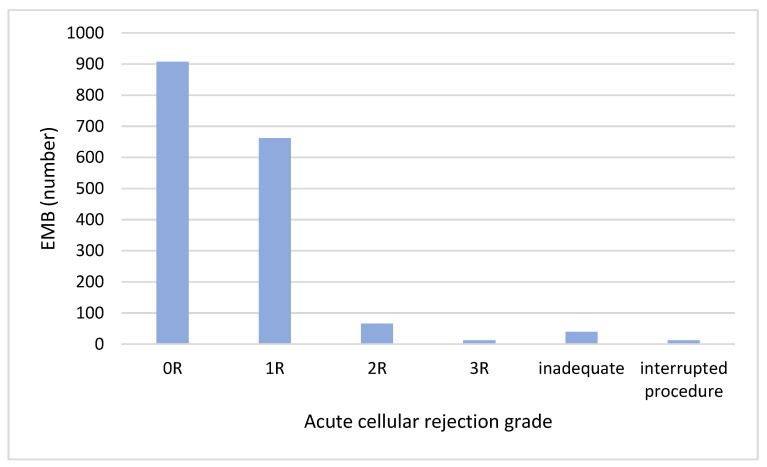
Acute cellular rejection grade of EMBs performed from 2012 to 2022.

**Figure 5:**
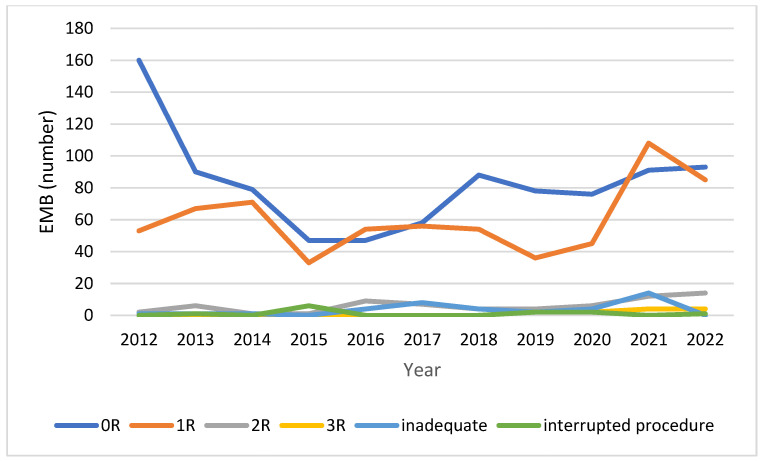
Time trend of acute cellular rejection grade from 2012 to 2022.

During the study period, 907 (53%) EMBs revealed ACR grade 0, 662 (39%) grade 1R, 66 (4%) grade 2R and 12 (1%) grade 3R; 39 (2%) EMBs provided inadequate samples for histological analysis and 12 (1%) EMBS were interrupted before sampling for patients’ discomfort or procedural complications (Figure 4). The temporal trend of ACR is showed in Figure 5.

**Figure 6:**
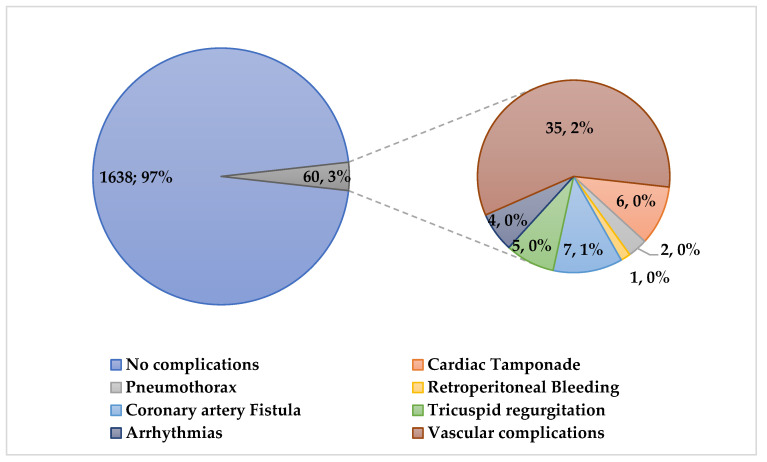
On the left: total number and percentage of uncomplicated and complicated EMBs recorded in the study population from 2012 to 2022. On the right: total number and percentage of major and minor complications recorded after EMB.

We recorded 60 (3.5%) complications, including 9 (0.5%) major complications (six cardiac tamponades, two pneumothorax and one retroperitoneal bleeding) and 51 (3%) minor complications (seven coronary artery fistulae, five de novo tricuspid regurgitation, four supraventricular arrythmias and thirty-five vascular access site complications) (Figure 6).

Details of total procedure-related complications and stratified for vascular access site for EMBs are provided in Table 2. No difference was found in total (38 [4%] vs. 22 [3%]; *p* = 0.16), major (6 [1%] vs. 3 [0.4%]; *p* = 0.65) and minor complications (32 [3%] vs. 19 [2%]; *p* = 0.23) between the TJ group and the TF group.

No difference was found in male sex, age at time of EMB and time from HT between complicated and not complicated EMBs (Table 3).

No difference was found in male sex and age between patients who had a complicated BEM between the TF and TJ approach; time from HT was significantly shorter in patients with a complicated EMB with a TJ approach (Table 4).

## 4. Discussion

In this retrospective single-center study, we showed that EMBs are a safe procedure with a complication rate less than 4%. These results are consistent with previously published reports. Yilmaz et al. reported on the procedural safety and diagnostic performance of left ventricular, right ventricular and biventricular EMBs in 755 patients with suspected myocarditis or non-ischemic cardiomyopathy [12]. The authors found a rate of major complications of 0.82% and a rate of minor complication of 2.2% to 5.1% for right ventricular EMBs [12]. Holzmann et al. analyzed the incidence of major and minor EMB procedure-related complications of a large cohort of 1919 retrospective and 496 prospective non-heart-recipient patients [13]. A total of 3048 EMB procedures were performed over a 11-year period via the right femoral vein. The authors found major complications in only 0.12% of the patients and minor complications in 5.7% of the patients [13]. Cooper et al. also reported a frequency of EMB complications of under 6% [14]. However, the rate of complications after EMBs reported by these authors refers to that mainly observed in patients diagnosed with either myocarditis or cardiomyopathy of unknown origin, not those with a HT. It has been reported that the risk of major complications is lower in HT recipients compared with non-HT patients (0.19% vs. 0.70%) [15]. Non-HT patients may suffer from acute or advanced heart failure with dilated ventricles and may be hemodynamically unstable at time of the EMB, with a higher risk of cardiac perforation, tamponade and malignant arrhythmias. A more recent report by Bermpeis et al. showed an overall complication rate of 4.1% in 1368 right and left ventricular biopsies performed for allograft rejection surveillance and diagnosis of cardiomyopathy from 2011 and 2021 [16]. Complications were more often observed in older, female and cardiomyopathy patients. In contrast with previous reports, a recent article analyzed the cost and use trends of EMBs between 2016 and 2019 in 8170 HT recipients and found that up to 25.2% of patients receiving an EMB experienced a post-procedure complication [17]. The most frequent complications were tricuspid valve regurgitation (45.6 to 49.7%), cardiac arrhythmia (23.8 to 26.2%) and atrioventricular block (4.8 to 9.7%) [17]. The higher rate of complications observed in this series could be probably due to tricuspid regurgitation, which is a complication frequently observed in HT recipients, because of the numerous biopsies performed for allograft rejection surveillance, in which the bioptome is repeatedly passed across the tricuspid valve and may damage both the valvular and sub-valvular apparatus [18]. Previous studies have shown that 47% of patients with new onset tricuspid valve regurgitation had evidence of chordal tissue in their myocardial specimens, suggesting that chordal damage resulting in flail leaflets is likely the mechanism of tricuspid valve regurgitation [19].

Tricuspid valve regurgitation represents the most common valvular complication following HT with a prevalence ranging from 19% to 84% [20]. In most cases, tricuspid valve regurgitation is mild and asymptomatic; however, up to 34% patients have symptomatic moderate to severe tricuspid valve regurgitation [21] and up to 5.8% of patients develop refractory symptoms requiring surgical correction [22]. Tricuspid valve regurgitation after HT has been associated with a reported increased mortality as high as 62.5%.

Numerous studies have demonstrated an association between the number of EMBs and the development of tricuspid valve regurgitation. Nguyen at al. reported no cases of severe tricuspid valve regurgitation in patients who have had fewer than 18 biopsies and a severe tricuspid valve regurgitation prevalence of 60% in patients with over 31 procedures. Following multivariate analysis, the authors found that only the total number of EMBs was an independent risk factor for tricuspid valve regurgitation severity [23]. In accordance with these reports, in our series the median number of EMBs for each patient was less than 10 and we observed only 0.3% of tricuspid valve regurgitation.

Another complication that is peculiar to heart recipients is the development of coronary artery fistulas. Coronary artery fistulas are rare coronary anomalies, affecting 0.1% to 0.2% of the population and are usually diagnosed incidentally during coronary angiography or noninvasive cardiac imaging [24]. The majority of coronary artery fistulas are congenital, but they can also represent an acquired disease following intracardiac device implantation, cardiac surgery, endomyocardial biopsy or direct chest trauma. Small coronary artery fistulas are generally asymptomatic and can close over time, while medium or large fistulas can enlarge and cause myocardial ischemia or cardiac chamber enlargement if left untreated [24]. In our series, only seven (0.4%) patients developed coronary fistulae that were incidentally diagnosed at follow-up annual coronary angiography and all had a benign evolution. In contrast to our results, previous studies have reported a higher incidence of coronary fistulas in heart recipients. Saraiva et al. found a 2.8% rate of coronary fistulae; all fistulae ended in the right ventricle and were small in size and asymptomatic, allowing a conservative approach [25]. Other series reported an even higher incidence of coronary fistulae up to 8% [26,27,28].

In our series, we did not find any difference in EMB-related complications between the TF and TJ approach; however, previous reports have highlighted that one obvious advantage of the trans-femoral approach is the absence of risk of pneumothorax or hemothorax. Furthermore, the Cordis bioptome used for TF EMBs is more flexible and less traumatic than the bioptome used for TJ EMBs [13]. Our results are consistent with those of a recent report showing no difference in EMB-related complications stratified for the access site (femoral artery, femoral vein and jugular vein) [16].

Procedural volume and operator expertise represent the most important determinants of risk of EMB-related complications. High-volume centers have a lower complication rate compared with low-volume centers, and high procedural volume has been identified as an independent predictor of a lower risk of major complications [29]. Ultrasound guidance for internal jugular central venous catheter placement has become the recommended best practice and has been shown to increase successful catheter placement and to reduce complications [30,31]. Likewise, ultrasound guidance of venous puncture when performing EMBs could reduce the incidence of vascular complications and pneumothorax. A recent report showed that surveillance EMBs have declined in the contemporary era, with a higher incidence of EMB complications compared with detected AR [32]. The authors reported that the risk of EMB complications was highest within 1 month after HT and suggested that surveillance EMB protocols in the contemporary era should be reevaluated [32]. Therefore, new less invasive strategies for detection of allograft rejection are required to reduce patients’ discomfort and procedural complications caused by EMB.

Cardiac magnetic resonance (CMR) is considered the gold-standard imaging modality for assessing cardiac morphology, ventricular volumes, systolic function and myocardial mass and it is also able to assess myocardial inflammatory changes including edema, hyperemia, capillary leak and irreversible injury [33]. A previous study demonstrated that a combined CMR approach using T2 mapping and extracellular volume quantification provided a high diagnostic accuracy for acute rejection diagnosis and could potentially decrease the number of routine EMBs [34]. Additionally, the results of the randomized trial published by Anthony et al. showed the feasibility of CMR-based surveillance for allograft rejection, reducing the potential complications associated with EMB-based surveillance [35].

Several biomarkers have been proposed to detect and monitor allograft rejection after HT.

The ST2/IL-33 pathway has been shown to be involved in the context of acute rejection after HT in both adults and pediatric heart recipients [36,37]. Serum sST2 levels significantly rise in the context of acute rejection showing a linear association with severity of acute rejection and decline after successful rejection therapy [38,39].

Other biomarkers include markers of allograft injury such as troponin and donor-derived cell-free DNA (dd-cfDNA) and markers of the inflammatory and allo-immune processes underlying allograft rejection such as AlloMap and microRNA (miRNA) [40]. A systematic review showed that cardiac troponins do not have sufficient specificity to diagnose acute cellular rejection in place of EMB. However, high-sensitivity troponin assays may have sufficient sensitivity and negative predictive value to exclude acute cellular rejection and limit the need for surveillance EMBs [41]. A large multicenter study found a strong correlation between dd-cfDNA and rejection and also demonstrated a rise in dd-cfDNA before the occurrence of biopsy-proven rejection and a drop following the treatment of rejection, findings that suggest the potential clinical utility of this biomarker [42]. As a result of these findings, the latest guidelines from the International Society for Heart and Lung Transplantation support the use of dd-cfDNA as a useful non-invasive biomarker to monitor and detect allograft rejection [43,44].

AlloMap is a non-invasive test based on the gene-expression profiling of peripheral blood mononuclear cells. An 11 gene real-time PCR test was derived from 252 candidate genes, converted into a score (0–40) and validated. The test detects accurately ACR ≥ 2R on the concomitant EMB. Patients > 1-year post-transplant with scores below 30 are unlikely to have grade ≥ 2R rejection [45]. Further studies have confirmed that AlloMap is not inferior to an EMB for rejection surveillance after HT and does not result in increased adverse outcomes but can reduce the number of EMBs performed [46,47,48].

MicroRNAs have been shown to be involved in gene expression regulation and in many biological processes, including immunological processes. Several studies have shown a role of miRNAs in the pathophysiology of cardiac allograft rejection and specific miRNA profiles in EMBs from patients with or without rejection [49]. However, although the results of these studies are very promising, randomized trials are still lacking and the use of miRNAs in clinical practice is not yet supported.

In vivo animal studies have proven the safety of a micro-biopsy device that could be used with a micro-catheter and minimize endocardial and valvular trauma [50]. However, whereas in a conventional EMB, the tissue samples are typically analyzed with conventional histology, for micro-biopsy samples, other techniques such as RNA-sequencing should be used as they require much smaller sample volumes. Specific gene expression signatures for transplant rejection have already shown promising results in human heart biopsies [51].

## 5. Limitations

This study has limitations due to the retrospective observational design where selection bias is unavoidable. In addition, this study covers a long period of time, and the post-transplant management of these patients could have changed over time. Therefore, the study results should be interpreted with caution.

## 6. Conclusions

Endomyocardial biopsy represents the gold standard for monitoring allograft rejection after HT. In our series, we recorded a total complication rate of 3.5% and a major complication rate of 0.5% with no difference between the TJ and the TF approach. Reported EMB complication rates have remained unchanged in the last decades, while the incidence of allograft rejection detected by EMBs has significantly reduced due to advances in immunosuppressive regimens. Alternative, less invasive strategies have shown their ability to discriminate between patients with and without allograft rejection and their use could minimize the number of routine EMBs, thus reducing procedural complications and patients’ discomfort. Future randomized controlled trials comparing surveillance EMBs to non-invasive strategies are necessary to evaluate whether our current practice of surveillance EMBs is still appropriate.

## Figures and Tables

**Table 1:** Characteristics of heart recipients included in the study.

Characteristics	Heart Recipients (n = 212)
Male sex, n (%)	158 (75%)
Age at time of HT, years	57 (49–65)
Dilated cardiomyopathy, n (%)	86 (41%)
Ischemic cardiomyopathy, n (%)	85 (40%)
Valvular cardiomyopathy, n (%)	11 (5%)
Myocarditis, n (%)	9 (4%)
Peri-partum cardiomyopathy	5 (2%)
Hypertrophic cardiomyopathy, n (%)	3 (1%)
Radiation- or chemotherapy-induced cardiomyopathy	3 (1%)
Arrhythmogenic right ventricular dysplasia	3 (1%)
Other cardiomyopathies	7 (3%)
Endomyocardial biopsy, n (interquartile range)	9 (2–12)

**Table 2:** Total, major and minor complications of EMBs and stratified for vascular access site used for EMB.

Characteristics	Total EMBs (n = 1698)	Trans-Jugular EMBs (n = 927)	Trans-Femoral EMBs (n = 771)	*p*
Male sex, n (%)	1309 (77%)	744 (80%)	565 (73%)	<0.001
Age at time of EMB, years	57 (49–63)	57 (50–64)	56 (47–63)	0.003
Time from HT, years	0.4 (0.1–0.9)	0.2 (0.1–0.5)	0.7 (0.3–2.8)	<0.001
Total Complications, n (%)	60 (3.5%)	38 (4%)	22 (3%)	0.16
Major complications, n (%)	9 (0.5%)	6 (1%)	3 (0.4%)	0.65
Cardiac tamponade n (%)	6 (0.4%)	4 (0.4%)	2 (0.3%)	0.55
Pneumothorax, n (%)	2 (0.1%)	2 (0.2%)	0	-
Retroperitoneal bleeding	1 (0.05%)	0	1 (0.1%)	-
Minor complications, n (%)	51 (3%)	32 (3%)	19 (2%)	0.23
Coronary artery fistulae, n (%)	7 (0.4%)	0	7 (1%)	-
De novo tricuspid regurgitation, n (%)	5 (0.3%)	3 (0.3%)	2 (0.3%)	0.8
Arrhythmias, n (%)	4 (0.2%)	3 (0.3%)	1 (0.1%)	0.4
Vascular access site, n (%)	35 (2%)	26 (3%)	9 (1%)	0.02
Hematoma, n (%)	13 (1%)	5 (1%)	8 (1%)	0.24
Accidental arterial puncture	22 (1%)	21 (2%)	1 (0.1%)	<0.001

**Table 3:** Characteristics of complicated and not complicated EMBs.

Characteristics	Total EMBs (n = 1698)	Not Complicated EMBs (n = 1638)	Complicated EMBs (n = 60)	*p*
Male sex, n (%)	1309 (77%)	1267 (77%)	42 (70%)	0.17
Age at time of EMB, years	57 (49–63)	57 (49–63)	57 (46–64)	0.78
Time from HT, years	0.4 (0.1–0.9)	0.4 (0.1–0.9)	0.3 (0.1–1.2)	0.9

**Table 4:** Characteristics of patients with EMB-related complications.

Characteristics	All (n = 60)	Trans-Jugular (n = 38)	Trans-Femoral (n = 22)	*p*
Male sex, n (%)	42 (70%)	27 (71%)	15 (68%)	0.8
Age at time of EMB, years	57 (46–64)	60 (47–65)	54 (46–60)	0.2
Time from HT, years	0.3 (0.1–1.2)	0.2 (0.1–0.5)	1.2 (0.5–14)	<0.001

## Data Availability

The data presented in this study are available on request from the corresponding author.
